# Transgender social inclusion and equality: a pivotal path to development

**DOI:** 10.7448/IAS.19.3.20803

**Published:** 2016-07-17

**Authors:** Vivek Divan, Clifton Cortez, Marina Smelyanskaya, JoAnne Keatley

**Affiliations:** 1 United Nations Development Programme Consultant, Delhi, India; 2 United Nations Development Programme, HIV, Health, and Development Group, New York, NY, USA; 3 United Nations Development Programme Consultant, New York, NY, USA; 4 University of California, San Francisco, Center of Excellence for Transgender Health, San Francisco, CA, USA

**Keywords:** Trans people health, trans people rights, sustainable development, HIV & development, SDGs, transgender

## Abstract

**Introduction:**

The rights of trans people are protected by a range of international and regional mechanisms. Yet, punitive national laws, policies and practices targeting transgender people, including complex procedures for changing identification documents, strip transgender people of their rights and limit access to justice. This results in gross violations of human rights on the part of state perpetrators and society at large. Transgender people's experience globally is that of extreme social exclusion that translates into increased vulnerability to HIV, other diseases, including mental health conditions, limited access to education and employment, and loss of opportunities for economic and social advancement. In addition, hatred and aggression towards a group of individuals who do not conform to social norms around gender manifest in frequent episodes of extreme violence towards transgender people. This violence often goes unpunished.

**Discussion:**

The United Nations Development Programme (UNDP) views its work in the area of HIV through the lens of human rights and advances a range of development solutions such as poverty reduction, improved governance, active citizenship, and access to justice. This work directly relates to advancing the rights of transgender people. This manuscript lays out the various aspects of health, human rights, and development that frame transgender people's issues and outlines best practice solutions from transgender communities and governments around the globe on how to address these complex concerns. The examples provided in the manuscript can help guide UN agencies, governments, and transgender activists in achieving better standards of health, access to justice, and social inclusion for transgender communities everywhere.

**Conclusions:**

The manuscript provides a call to action for countries to urgently address the violations of human rights of transgender people in order to honour international obligations, stem HIV epidemics, promote gender equality, strengthen social and economic development, and put a stop to untrammelled violence.

## Introduction

Those who have traditionally been marginalized by society and who face extreme vulnerability to HIV find that it is their marginalization – social, legal, and economic – which needs to be addressed as the highest priority if a response to HIV is to be meaningful and effective. Trans people's experiences suggest that although HIV is a serious concern for those who acquire it, the suffering it causes is compounded by the routine indignity, inequity, discrimination, and violence that they encounter. Trans people, and particularly trans women, have articulated this often in the context of HIV [1].

For a reader who is not trans, imagine a world in which the core of your being goes unrecognized – within the family, if and when you step into school, when you seek employment, or when you need social services such as health and housing. You have no way to easily access any of the institutions and services that others take for granted because of this denial of your existence, worsened by the absence of identity documents required to participate in society. Additionally, because of your outward appearance, you may be subject to discrimination, violence, or the fear of it. In such circumstances, how could you possibly partake in social and economic development? How could your dignity and wellbeing – physical, mental, and emotional – be ensured? And how could you access crucial and appropriate information and services for HIV and other health needs?

Trans people experience these realities every day of their lives. Yet, like all other human beings, trans people have fundamental rights – to life, liberty, equality, health, privacy, speech, and expression [2], but constantly face denial of these fundamental rights because of the rejection of the trans person's right to their gender identity. In these circumstances, there can be no attainment of the goal of universal equitable development as set out in the 2030 Agenda for Sustainable Development [3], and no effort to stem the tide of the HIV epidemic among trans people can succeed if their identity and human rights are denied.

## Discussion

### The human rights gap – stigma, discrimination, violence

The ways in which marginalization impacts a trans person's life are interconnected; stigma and transphobia drive isolation, poverty, violence, lack of social and economic support systems, and compromised health outcomes. Each circumstance relates to and often exacerbates the other [4].

Trans people who express their gender identity from an early age are often rejected by their families [5]. If not cast out from their homes, they are shunned within households resulting in lack of opportunities for education and with no attempts to ensure attention to their mental and physical health needs. Those who express their gender identities later in life often face rejection by mainstream society and social service institutions, as they go about undoing gender socialization [6]. Hostile environments that fail to understand trans people's needs threaten their safety and are ill-equipped to offer sensitive health and social services.

Such discriminatory and exclusionary environments fuel social vulnerability over a lifetime; trans people have few opportunities to pursue education, and greater odds of being unemployed, thereby experiencing inordinately high levels of homelessness [6] and poverty [7]. Trans students experience resentment, prejudice, and threatening environments in schools [8], which leads to significant drop-out rates, with few trans people advancing to higher education [9].

Workplace-related research on lesbian, gay, bisexual, and trans (LGBT) individuals reveals that trans workers are the most marginalized and are excluded from gainful employment, with discrimination occurring at all phases of the employment process, including recruitment, training opportunities, employee benefits, and access to job advancement [10]. This environment inculcates pessimism and internalized transphobia in trans people, discouraging them from applying for jobs [11]. These extreme limitations in employment can push trans people towards jobs that have limited potential for growth and development, such as beauticians, entertainers or sex workers [12]. Unemployment and low-paying or high risk and unstable jobs feed into the cycle of poverty and homelessness. When homeless trans people seek shelter, they are housed as per their sex at birth and not their experienced gender, and are subject to abuse and humiliation by staff and residents [13]. In these environments, many trans people choose not to take shelter [14].

Legal systems often entrench this marginalization, feed inequality, and perpetuate violence against trans people. All people are entitled to their basic human rights, and nations are obligated to provide for these under international law, including guarantees of non-discrimination and the right to health [2]; however, trans people are rarely assured of such protection under these State obligations.

Instead, trans people often live in criminalized contexts – under legislation that punishes so-called unnatural sex, sodomy, buggery, homosexual propaganda, and cross-dressing [12] – making them subject to extortion, abuse, and violence. Laws that criminalize sex work lead to violence and blackmail from the police, impacting trans women involved in this occupation [15]. Being criminalized, trans people are discouraged from complaining to the police, or seeking justice when facing violence and abuse, and perpetrators are rarely punished. When picked up for any of the aforementioned alleged crimes or under vague “public nuisance” or “vagrancy” laws, their abuse can continue at the hands of the police [16] or inmates in criminal justice systems that fail to appropriately respond to trans identities.

The transphobia that surrounds trans people's lives fuels violence against them. Documentation over the last decade reveals the disproportionate extent to which trans people are murdered, and the extreme forms of torture and inhuman treatment they are subject to [16–18]. When such atrocities are perpetrated against trans people, governments turn a blind eye. Trans sex workers are particularly vulnerable to brutal police conduct including rape, sometimes being sexually exploited by those who are meant to be protectors of the law [15]. In these circumstances, options to file complaints are limited and, when legally available channels do exist, trans complainants are often ignored [19].

These experiences of severe stigma, marginalization, and violence by families, communities, and State actors lead to immense health risks for trans people, including heightened risk for HIV, mental health disparities, and substance abuse [20,21]. However, most health systems struggle to function outside the traditional female/male binary framework, thereby excluding trans people [22]. Health personnel are often untrained to provide appropriate services on HIV prevention, care, and treatment or information on sexual and reproductive health to trans people [20,23]. HIV voluntary counselling and testing facilities and antiretroviral therapy (ART) sites intimidate trans people due to prior negative experiences with medical staff [21,24,25]. Additionally, when trans women test HIV positive, they are wrongly reported as men who have sex with men [4]. Consequently, testing rates in trans communities are low [26], which serves to disguise the serious burden of HIV among trans people and perpetuates the lack of investment in developing trans-sensitive health systems. The economic hardships that trans people face due to their inability to participate in the workforce further complicate access to HIV, mental health, and gender-affirming health services. In short, hostile social and legal environments contribute to health gaps, and public health systems that are unresponsive to the needs of trans people.

In addition, understanding of trans people's concerns around stigma, discrimination, and violence, related as they are to gender identity, is often limited due to their being combined with lesbian, gay, and bisexual sexual orientation issues. However, trans people's human rights concerns, grounded in their gender identity, are inherently different and necessitate their own set of approaches.

### Imperatives for trans social inclusion

In order to overcome the human rights barriers trans people confront, certain measures are imperative and should be self-evident, given the standards that States are obliged to provide under international law to all human beings. Paying attention to these is key to effectively addressing the systemic marginalization that trans people experience. Such action can have immeasurable benefits, including the full participation of trans people in human development processes as well as positive health and HIV outcomes. For trans people, the change must begin with the most fundamental element – acknowledgement of their gender identity.

#### The right to gender recognition

For trans people, their very recognition as human beings requires a guarantee of a composite of entitlements that others take for granted – core rights that recognize their legal personhood. As the Global Commission on HIV and the Law pointed out, “In many countries from Mexico to Malaysia, by law or by practice, transgender persons are denied acknowledgment as legal persons. A basic part of their identity – gender – is unrecognized” [19]. This recognition of their gender is core to having their inherent dignity respected and, among other rights, their right to health including protection from HIV. When denied, trans people face severe impediments in accessing appropriate health information and care.

Recognizing a trans person's gender requires respecting the right of that person to identify – irrespective of the sex assigned to them at birth – as male, female, or a gender that does not fit within the male–female binary, a “third” gender as it were, as has been expressed by many traditionally existing trans communities such as *hijras* in India [27]. This is an essential requirement for trans people to attain full personhood and citizenship. The guarantee of gender recognition in official government-issued documents – passports and other identification cards that are required to open bank accounts, apply to educational institutions, enter into housing or other contracts or for jobs, to vote, travel, or receive health services or state subsidies – provides access to a slew of activities that are otherwise denied while being taken for granted by cisgender people.^1^ Such recognition results in fuller civic participation of and by trans people. It is a concrete step in ensuring their social integration, economic advancement, and a formal acceptance of their legal equality. It can immeasurably support their empowerment and act as an acknowledgement of their dignity and human worth, changing the way they are perceived by their families, by society in general, and by police, government actors, and healthcare personnel whom they encounter in daily life. UN treaty bodies have acknowledged this vital right of trans people to be recognized. The UN High Commissioner for Human Rights has recommended that States “facilitate legal recognition of the preferred gender of transgender persons and establish arrangements to permit relevant identity documents to be reissued reflecting preferred gender and name, without infringements of other human rights” [28].

#### Freedom from violence & discrimination

Systemic strategies to reduce the violence against trans people need to occur at multiple levels, including making perpetrators accountable, facilitating legal and policy reform that removes criminality, and general advocacy to sensitize the ill-informed about trans issues and concerns. Strengthening the capacity of trans collectives and organizations to claim their rights can also act as a counter to the impunity of violence. When trans people are provided legal aid and access to judicial processes, accountability can be enforced against perpetrators. Sensitizing the police to make them partners in this work can be crucial. When political will is absent to support such attempts in highly adverse settings, trans organizations and allies can consider using international human rights mechanisms, such as shadow reports made to UN human rights processes like the Universal Periodic Review, to bring focus to issues of anti-trans violence and other human rights violations against trans people.

Providing equal access to housing, education, public facilities and employment opportunities, and developing and implementing anti-discrimination laws and policies that protect trans people in these contexts, including guaranteeing their safety and security, are essential to ensure that trans individuals are treated as equal human beings.

#### The right to health

For trans people, their right to health can only be assured if services are provided in a non-stigmatizing, non-discriminatory, and informed environment. This requires working to educate the healthcare sector about gender identity and expression, and zero tolerance for conduct that excludes trans people. Derogatory comments, breaches of confidentiality from providers, and denial of services on the basis of gender identity or HIV status are some of the manifestations of prejudice. The right to non-discrimination that is guaranteed to all human beings under international law must be enforced against actions that violate this principle in the healthcare system. Yet, a multi-pronged approach that supports this affirmation of trans equality together with a sensitized workforce that is capable of delivering gender-affirming surgical and HIV health services is necessary.

Building on the commitments made by the UN General Assembly in response to the HIV epidemic [29], the World Health Organization (WHO) developed good practice recommendations in relation to stigma and discrimination faced by key populations, including trans people [30]. These recommendations urge countries to introduce rights-based laws and policies and advise that, “Monitoring and oversight are important to ensure that standards are implemented and maintained.” Additionally, mechanisms should be made available “to anonymously report occurrences of stigma and/or discrimination when [trans people] try to obtain health services” [30].

Fostering stigma-free environments has been successfully demonstrated – where partnerships between trans individuals and community health nurses have improved HIV-related health outcomes [31], or where clinical sites welcome trans people and conduct thorough and appropriate physical exams, manage hormones with particular attention to ART, and engage trans individuals in HIV education [32].

### Advancing trans human rights and health

For all the challenges faced by trans people in the context of their human rights and health, promising interventions and policy progress have shown that positive change is possible, although this must be implemented at scale to have significant impact. Change has occurred due to the efforts of trans advocates and human rights champions, often in critical alliances with civil society supporters as well as sensitized judiciaries, legislatures, bureaucrats, and health sector functionaries.

Key strides have been made in the context of gender recognition in some parts of the world. In the legislatures, this trend began in 2012 with Argentina passing the *Gender Identity and Health Comprehensive Care for Transgender People Act*, which provided gender recognition to trans people without psychiatric, medical, or judicial evaluation, and the right to access free and voluntary transitional healthcare [33,34]. In 2015, Malta passed the *Gender Identity, Gender Expression and Sex Characteristics Act*, which provides a self-determined, speedy, and accessible gender recognition process. The law protects against discrimination in the government and private sectors. It also de-pathologizes gender identity by stating that people “shall not be required to provide proof of a surgical procedure for total or partial genital reassignment, hormonal therapies or any other psychiatric, psychological or medical treatment.” It presumes the capacity of minors to exercise choice in opting for gender reassignment, while recognizing parental participation and the minor's best interests. It stipulates the establishment of a working group on trans healthcare to research international best practices [35]. Pursuant to its passing the Maltese Ministry of Education working with activists also developed policy guidelines to accommodate trans, gender variant, and intersex children in the educational system [36]. Other countries, such as the Republic of Ireland and Poland, have also passed gender identity and gender expression laws, albeit of varying substance but intended to recognize the right of trans people to personhood [37,38]. Denmark passed legislation that eliminated the coercive requirement for sterilization or surgery as a prerequisite to change legal gender identity [39].

Trans activists and allies have also used the judicial process to claim the right to gender recognition. In South Asia, claims to recognition of a gender beyond the male–female binary have been upheld – in 2007, the Supreme Court of Nepal directed the government to recognize a third gender in citizenship documents in order to vest rights that accrue from citizenship to *metis* [40]; in Pakistan, the Supreme Court directed the government to provide a third gender option in national identity cards for trans people to be able to vote [41]; in 2014, the Indian Supreme Court passed a judgement directing the government to officially recognize trans people as a third gender and to formulate special programmes to support their needs [42]. These developments in law, while hopeful, are too recent to yet discern any resultant trends in improvements in trans peoples’ lives, more broadly.

More localized innovative efforts have also been made by trans organizations to counter violence, stigma, and discrimination. For instance in South Africa, Gender DynamiX, a non-governmental organization worked with the police to change the South African Police Services’ standard operating procedures in 2013. The procedures are intended to ensure the safety, dignity, and respect of trans people who are in conflict with the law, and prescribe several trans-friendly safeguards – the search of trans people as per the sex on their identity documents, irrespective of genital surgery, and detention of trans people in separate facilities with the ability to report abuse, including removal of wigs and other gender-affirming prosthetics. Provision is made for implementation of the procedures through sensitization workshops with the police [43]. In Australia, the Transgender Anti-Violence Project was started as a collaboration between the Gender Centre in Sydney and the New South Wales Police Force, the City of Sydney and Inner City Legal Centre in 2011. It provides education, referrals, and advocacy in relation to violence based on gender identity, and support for trans people when reporting violence, assistance in organizing legal aid and appearances in court [44].

Measures have also been taken to tackle discrimination faced by trans people, in recognition of their human rights – in 2015, Japan's Ministry of Education ordered schools to accept trans students according to their preferred gender identity [45]; in 2014 in Quezon City, the Philippines the municipal council passed the “Gender Fair City” ordinance to ensure non-discrimination of LGBT people in education, the workplace, media depictions, and political life. This law prohibits bullying and requires gender-neutral bathrooms in public spaces and at work [46]; in Ecuador, Alfil Association worked on making healthcare accessible to trans people, including training and sensitization meetings for health workers and setting up a provincial health clinic for trans people in collaboration with the Ministry of Health, staffed by government physicians who had undergone the training; and Transbantu Zambia set up a small community house providing temporary shelter for trans people, assisting them in difficult times or while undergoing hormone therapy. Similar housing support has been provided by community organizations with limited resources in Jamaica and Indonesia.^2^

## Conclusions

### Towards sustainable development: time for change

Although there are other examples of human rights progress for trans people, much of this change is isolated, non-systemic, and insufficient. Trans people continue to live in extremely hostile contexts. What is required is change and progress at scale. The international community's recent commitment towards Sustainable Development Goals (SDGs) presents an opportunity to catalyze and expand positive interventions [3].

Preventing human rights violations and social exclusion is key to sustainable and equitable development. This is true for trans people as much as other human beings, just as the achievement of all 17 SDGs is of paramount importance to all people, including trans people. Of these SDGs, the underpinning support for trans people's health and human rights is contained in SDG 3 –“Ensure healthy lives and promote well-being for all at all ages,” SDG 10 – “Reduce inequality within and among countries,” and SDG 16 – “Promote peaceful and inclusive societies for sustainable development, provide access to justice for all and build effective, accountable and inclusive institutions at all levels.”

The SDGs are guided by the UN Charter and grounded in the Universal Declaration of Human Rights. They envisage processes that are “people-centered, gender-sensitive, respect human rights and have a particular focus on the poorest, most vulnerable and those furthest behind” and a “just, equitable, tolerant, open and socially inclusive world in which the needs of the most vulnerable are met” [3]. They reiterate universal respect for human rights and dignity, justice and non-discrimination, and a world of equal opportunity permitting the full realization of human potential for all irrespective of race, colour, sex, language, religion, political or other opinion, national or social origin, property, birth, disability, or *other status*. The relationship between the SDGs and trans people's concerns has been robustly articulated in the context of inclusive development [47].

UN Member States have unequivocally agreed to this new common agenda for the immediate future. The SDGs demand an unambiguous, farsighted, and inclusive demonstration of political will. Their language clearly reflects the most urgent needs of trans people, for whom freedom from violence and discrimination, the right to health and legal gender recognition are inextricably linked.

Specifically in regard to trans people, the SDGs are a call to immediate action on several fronts: governments need to engage with trans people to understand their concerns, unequivocally support the right of trans people to legal gender recognition, support the documentation of human rights violations against them, provide efficient and accountable processes whereby violations can be safely reported and action taken, guarantee the prevention of such violations, and ensure that the whole gamut of robust health and HIV services are made available to trans people. Only then can trans people begin to imagine a world that respects their core personhood, and a world in which dignity, equality, and wellbeing become realities in their lives.

## Footnotes

1 Cisgender people identify and present in a way that is congruent with their birth-assigned sex. Cisgender males are birth-assigned males who identify and present themselves as male.

2 These illustrations are based on information gathered in the process of developing a tool to operationalize the Consolidated Guidelines on HIV prevention, diagnosis, treatment and care for key populations (WHO, 2014), through interviews with and questionnaires sent to trans activists. See also reference 31.
